# Parks and Green Areas Are Associated with Decreased Risk for Hyperlipidemia

**DOI:** 10.3390/ijerph13121205

**Published:** 2016-12-03

**Authors:** Hye-Jin Kim, Jin-Young Min, Hyun-Jin Kim, Kyoung-Bok Min

**Affiliations:** 1 School of Public Health, Seoul National University, Seoul 08826, Korea; okimhj@snu.ac.kr; 2 Institute of Health and Environment, Seoul National University, Seoul 08826, Korea; yaemin00@snu.ac.kr (J.-Y.M.); honey-hj@hanmail.net (H.-J.K.); 3 Department of Preventive Medicine, College of Medicine, Seoul National University, 103 Daehak-ro, Jongno-gu, Seoul 110-799, Korea

**Keywords:** natural environment, cardiovascular disease, physical activity, stress, lipid

## Abstract

This study aimed to investigate the association between parks and green areas and hyperlipidemia in adults with groups stratified by moderate physical activity as a behavioral modification using the 2009 Korean Community Health Survey data and 212,584 participants enrolled in this study. The geographical codes of study participants were all matched on the basis of the amount of parks and green areas in each administrative district. Compared with participants living in the highest quartile of parks and green areas (Quartile 4), those living in the lowest quartile of green and park area (Quartile 1) were at an increased risk of physician-diagnosed hyperlipidemia and hyperlipidemia currently under treatment. Participants in the lowest quartile of parks and green areas were likely not to engage in any moderate physical activity. After classifying hyperlipidemia risk depending on the presence of moderate physical activity, those participating in moderate physical activity were less likely to have hyperlipidemia in all quartiles of parks and green areas than those not engaging in moderate physical activity. We found that parks and green areas were associated with decreased hyperlipidemia risk. Physical activity, which may benefit from the presence of parks and green areas, may reduce hyperlipidemia risk.

## 1. Introduction

Cardiovascular disease (CVD) is a major public health problem and a leading cause of death and disability worldwide [1]. The pathogenic mechanisms may vary depending on the different forms of CVD (e.g., stroke or heart attack); however, an unhealthy diet, physical inactivity, smoking, excessive alcohol consumption, hypertension, elevated glucose levels/diabetes, and elevated cholesterol levels are established risk factors for CVD [1]. 

Recent reports have suggested that parks and other green environments are beneficial for cardiovascular health [2,3,4,5]. Many studies have showed that exposure or accessibility to a green space was associated with decreased risk of coronary heart disease, stroke, and circulatory mortality [6,7,8,9,10,11,12]. The benefits of parks and green spaces in mitigating behavioral and environmental risks of CVD include increased physical activity, alleviated psychosocial stress, increased social interaction, and reduced exposures to air pollution, noise, and heat waves [2,3,4,5,13,14]. Indeed, people living in greener environments had reduced risk for intermediate clinical variables of CVD, such as diabetes [15,16] and high blood pressure (BP) [17].

Hyperlipidemia is a critical risk factor for developing CVD [18,19]. Hyperlipidemia control is considered to be an initial target for reducing cardiovascular risk [20,21]. Given that parks and green spaces reduce behavioral and environmental risk factors for CVD, they may also help alleviate hyperlipidemia by reducing triglyceride (TG) levels, increasing high-density lipoprotein (HDL) cholesterol levels, and decreasing low-density lipoprotein (LDL) cholesterol to HDL ratios, thereby improving lipid profiles. Specifically, increased physical activity is associated with a positive modification in the lipid profile caused by decreased lipid peroxidation products and oxidative stress markers [22]. In addition, people living in small green areas are associated with an increased hyperlipidemia risk caused by increased intake of sweet and high-fat foods because of psychosocial stress [23], as well as changes in blood lipid markers due to increased exposure to air pollution [24]. However, to our knowledge, no research has addressed this association. 

In this study, we investigated the association between parks and green areas and hyperlipidemia in adults using groups stratified by moderate physical activity, as a behavioral modification in Korea. 

## 2. Materials and Methods 

### 2.1. Data Source and Study Population

Data were obtained from the 2009 Korean Community Health Survey (KCHS), which is a population-based cross-sectional study that was designed to collect nationwide epidemiological data for adults aged 19 years and older at the community level. Thirty-six universities and 253 community-based health centers were involved in this study. Well-trained investigators visited each individual’s household, and the subject’s health-related information was gathered through face-to-face interviews. A total of 230,715 adults participated in the 2009 KCHS. Of these, we excluded 81 individuals who had missing phenotypic information on hyperlipidemia-related questions. In addition, 18,050 individuals with missing information on various variables such as age, sex, stress, a history of diabetes, body mass index (BMI), and physical activity were excluded from analysis. This study therefore included a total of 212,584 individuals.

### 2.2. Exposure Measures: Park and Green Areas

From the Korean Statistical Information Service, we used the 2009 nationwide data that detailed the amount of parks and green areas (m^2^) per capita in 200 administrative districts to evaluate the level of exposure to parks and green areas for each sample. The average size of these administrative areas was 623.15 km^2^. The geographical codes of each subject were used to associate individuals with the level of parks and green areas from each administrative district. The amount of parks and green areas was classified into four levels using quartiles: Quartile 1 (≤14.90 m^2^/capita), Quartile 2 (14.90–22.40 m^2^/capita), Quartile 3 (22.41–33.30 m^2^/capita), and Quartile 4 (≥33.31 m^2^/capita).

### 2.3. Phenotypes

The outcome variables included the existence and treatment of hyperlipidemia (when participants had a history of hyperlipidemia) using self-reported questionnaires. The presence of hyperlipidemia was determined by asking respondents if they had been diagnosed with hyperlipidemia by a physician. Treatment was coded as a dichotomous variables (YES or NO) depending on whether participants were being treated for hyperlipidemia at the time of the survey. 

Other variables of interest included demographic characteristics and health and behavioral information such as age, sex, marital status, education, monthly income, smoking, alcohol intake, a history of diabetes, and self-reporting stress. Job categories included (1) white-collar jobs, including managers and professionals; (2) pink-collar jobs, including clerical and jobs; (3) military jobs, including professional soldiers; and (4) no economic category, including unemployed individuals and students. BMI (kg/m^2^) was calculated by the self-reported weights and heights. The extent of moderate physical activity was determined based on the number of days per week on which individuals participated in moderate activities (i.e., leisure sports, including swimming, tennis, volleyball, badminton, and ping-pong, etc.) for at least 10 min. Individuals with ≥1 day per week of moderate physical activity were classified into the moderate physical activity group. Individuals were also asked the average time for which they participated in such activities. The time of moderate physical activity was classified into four levels using quartiles: Quartile 1 (<60 min/week), Quartile 2 (60–89 min/week), Quartile 3 (90–170 min/week), and Quartile 4 (≥170 min/week). 

### 2.4. Statistical Analysis

Differences in the characteristics of the participants based on the presence of physician-diagnosed and current treatment of hyperlipidemia were evaluated using the chi-square test. To find the association between hyperlipidemia and parks and green areas, we performed unadjusted and multivariate-adjusted logistic regression analyses to identify the association between hyperlipidemia and parks and green areas. The odds ratio (OR) and the corresponding 95% confidence interval (CI) of hyperlipidemia were generated by comparing the quartile with the highest number of parks and green areas (Quartile 4) as the reference group. The regression models were adjusted for age, sex, marital status, education, monthly income, jobs, smoking status, alcohol drinking, a history of diabetes mellitus, BMI, self-reporting stress, and moderate physical activity. We also categorized moderate activity time per week into either doing activity (YES) or not (NO). Furthermore, we classified doing activity (YES) group into Quartiles and graphed the percentage of time of moderate physical activity (no activity, level 1, level 2, level 3, level 4) by the quartile of park and green areas. Finally, we calculated the OR and 95% CI for hyperlipidemia across the quartiles of parks and green areas stratified for moderate physical activity (either NO vs. YES). All statistical analyses were conducted using SAS 9.2 software (SAS Institute, Cary, NC, USA), and statistical significance was evaluated at a significance level of 0.05 (*p* < 0.05). 

## 3. Results

Table 1 shows the characteristics of the study population based on the quartile of parks and green areas. We found that the proportion of participants who are older (≥60 years), married, have blue-collar jobs, have lower level of education (elementary or less), lower income (≤2000), low self-reported stress, and perform highest level of moderate activity (level 4) were likely to live in the highest quartile (Quartile 4) of parks and green areas. On the other hand, participants who are younger (20–29), have white-collar jobs, higher level of education (college or higher), higher income (≥3501), high self-reported stress, and do not engage in moderate physical activity were likely to live in the lowest quartile (Quartile 1) of green areas. All variables were significantly different (*p* < 0.001) among the four quartiles of parks and green areas.

**Figure 1:**
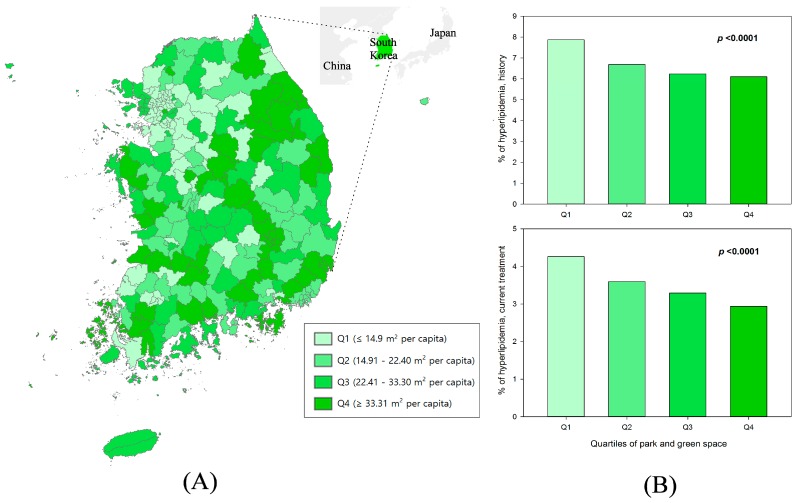
Distribution of park and green areas (m^2^) per capita (**A**) and percentages (%) of hyperlipidemia against the quartiles of parks and green areas (**B**).

Figure 1 shows the prevalence (%) of hyperlipidemia by the quartiles of parks and green areas. Participants living in the lowest quartiles of parks and green areas had the highest levels of hyperlipidemia, for both those physician-diagnosed and those currently receiving treatment. Overall, prevalence decreased as the quartiles of parks and green areas increased.

Table 2 shows the OR (95% CI) of hyperlipidemia with respect to both physician-diagnosed participants and those receiving treatment against the quartile of parks and green areas. We conducted a series of ordered logistic regression analyses. The OR indicated that the risk of both physician-diagnosed hyperlipidemia and hyperlipidemia currently under treatment was lower in the highest quartile of parks and green areas (Quartile 4) compared with the lowest quartile of parks and green areas (Quartile 1). A significant association between quartiles of parks and green areas and hyperlipidemia (physician-diagnosed and current treatment) were detected after controlling for demographic variables (model 1), smoking and alcohol (model 2), and physical activity (model 3). Compared with the unadjusted OR, fully adjusted OR of diagnosed hyperlipidemia and hyperlipidemia currently under treatment was significantly decreased with descending quartiles of parks and green areas.

**Figure 2:**
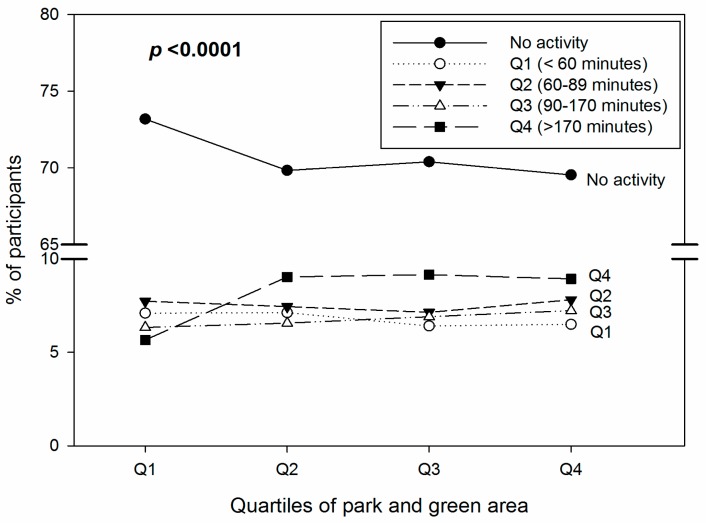
Percentages (%) of time of moderate physical activity (per week) by the quartiles of parks and green areas.

Figure 2 shows the percentages (%) of moderate activity time against the quartile of parks and green areas. Participants living in the lowest park and green areas (Quartile 1) comprised the highest percentages of those participating in no moderate physical activity or brief activities compared with those living in other quartiles (Quartile 2–Quartile 4).

Table 3 shows OR (95% CI) of hyperlipidemia by the quartiles of parks and green areas depending on the presence of moderate physical activity adjusted for demographic variables (age, sex, marriage, education, monthly income, and job categories) and health-related behaviors (alcohol and smoking). As examined in Table 2, participants living in the lower quartiles of parks and green areas were likely to have physician-diagnosed hyperlipidemia whether they engaged in moderate physical activity (YES) or not (NO). After stratification by physical activity (YES or NO), overall groups with no moderate physical activity (NO) were more likely to have a diagnosis of hyperlipidemia than the moderate physical activity group (YES). For example, in the moderate physical activity (NO) group, compared with Quartile 4, ORs of diagnosed hyperlipidemia in Quartile 1 were 1.25 (95% CI, 1.18–1.33). In the moderate physical activity YES group, ORs of diagnosed hyperlipidemia in Quartile 1 were 1.19 (95% CI, 1.08–1.30). In the same manner, subjects living in lower parks and green areas were likely to have hyperlipidemia currently under treatment whether there was moderate physical activity (YES) or not (NO). After stratification by physical activity (YES or NO), the moderate physical activity (NO) group was more likely to have hyperlipidemia currently being treated than the moderate physical activity group (YES). For example, in the moderate physical activity NO group, compared with Quartile 4, ORs of hyperlipidemia currently under treatment in Quartile 1 were 1.49 (95% CI, 1.37–1.62). In the moderate physical activity YES group, ORs of hyperlipidemia currently under treatment in Quartile 1 were 1.35 (95% CI, 1.18–1.55).

## 4. Discussion

We found that parks and green areas were associated with decreased hyperlipidemia risk in Korean adults. After adjusting for potential covariates, participants living in the lowest quartile of parks and green areas (Quartile 1) had significantly increased risk for physician-diagnosed hyperlipidemia, whether treated or not, compared with those living in the highest quartile of parks and green areas (Quartile 4). Participants living in the lowest park and green areas were likely to not engage in any moderate physical activity, which in itself increased hyperlipidemia (physician-diagnosed and currently under treatment) risk. This association was present for all four quartiles of parks and green areas in individuals who participated in moderate physical activity. This finding suggests that more parks and green areas would help lower hyperlipidemia risk. Furthermore, improving moderate physical activity in green areas may be associated with decreased hyperlipidemia risk, although to a lesser extent.

No studies have yet analyzed the association of parks and green areas with hyperlipidemia. However, a low hyperlipidemia risk is expected to be associated with parks and green areas, as it has previously been shown that green environments favorably affect clinical intermediate variables for cardiovascular risks, including hypertension and diabetes [15,16,17]. People residing in areas with neighborhood green spaces and those having high accessibility to green spaces had a lower diabetes risk than their counterparts [15,16]. Risk reduction for systolic BP elevation and hypertension were also associated with high quality green spaces [17]. In addition, residential proximity to city parks appears to have a beneficial impact of BP in early pregnancy, indicating that odds of normal BP or high-normal BP for every 300 m increase with the distance from green spaces by 9% and 14%, respectively [25].

The question is therefore how parks and green area positively affect cardiovascular health? Several potential mechanisms have been suggested to explain the association between parks and green areas and better health from Ulrich’s psych evolutionary building theory. For example, green areas offer places for physical activity, and encourage residents to be physically active [26,27]. Vegetation in green areas reduces air pollution, eliminates substances that are harmful to the human body [3], and reduces exposure to noise [28] and extreme heat [29]. Although we could not validate all above-mentioned potential mechanisms, the cumulative positive effects of green areas may potentially support our study results, by lowering LDL cholesterol of people living in higher parks and green areas.

Particularly, physical activity, one significant contributor factor, was demonstrated in our analysis. The results showed that participants living in areas with more parks and green spaces tended to engage in more moderate activity than their counterparts. Furthermore, the participants that engaged in moderate physical activity had relatively lower changes of having both physician-diagnosed hyperlipidemia and hyperlipidemia currently under treatment than those who did not engage in moderate physical activity. Admittedly, identifying the thresholds for the beneficial effect of physical activity on lipid metabolism is difficult. However, several studies have demonstrated that physical activity can favorably alter blood lipid profiles [30,31,32]. Indeed, Voulgari et al. reported that physical activity reduced the risk of CVD-related disorders, such as hypertension, diabetes mellitus, and hyperlipidemia [33]. A study by Bakheet et al. reported that regular moderate physical activity lowered the total mortality of the elderly, and had a positive effect on coronary heart disease prevention and lipid blood levels [34]. Taken together, residential environments with access to green spaces not only decrease hyperlipidemia risk directly but also encourage physical activity, which may help to induce desirable changes in lipid profile.

This is the first study to demonstrate the association between parks and green areas and hyperlipidemia, implicating physical activity as a mediator in this association. We analyzed a large data set from a nationwide sample and used an objective measurement of parks and green areas based on national statistical data. However, several limitations should be considered. Firstly, there were no data to measure the proximity or characteristics of each park and green area. Specifically, green area per capita could not account for individual exposure to green areas because of the large administrative unit. In addition, despite accuracy in measurement of parks and green areas, we could not consider specific qualities, such as safety, accessibility to the green environments and usage, and features and people’s perception of green spaces. Secondly, this study had a cross-sectional design wherein causal relationships cannot be directly ascertained. For example, people with healthier life styles might be more likely to live in green areas. We could not control the direction of causation due to the lack of data on health information (e.g., diet) or a negative control that associated with hyperlipidemia but not with green areas. Thirdly, the KCHS included only self-reported data, which has the disadvantage of recalling bias or misclassification in health and behavioral information. For instance, there would be a lack of objectivity in information on hyperlipidemia because it was self-reported, potentially leading to inconsistencies compared with the opinion of a clinical specialist. Finally, there would be a residual confounding effect by other factors that we have not controlled for in our models. Specifically, our results could be potentially biased in terms of diagnosis and treatment of hyperlipidemia depending on the health access of each individual (e.g., physician visits, screening, health information, and health care).

## 5. Conclusions

In conclusion, we observed that a higher exposure to parks and green areas were significantly associated with a lower risk of hyperlipidemia in Korean adults who were either diagnosed by a physician or receiving treatment. Physical activity, which may benefit from the presence of parks and green areas, may be associated with decreased hyperlipidemia risk. Although further studies are needed to clarify this, maintaining and promoting more parks and green spaces may be an important asset for better cardiovascular health.

## Figures and Tables

**Table 1:** Characteristics of the study population based on quartiles of parks and green area (m^2^ per capita) *n* (%).

	Quartile 1 (≤14.9)		Quartile 2 (14.91–22.4)		Quartile 3 (22.41–33.3)		Quartile 4 (≥33.31)		
	(*n* = 68,192)		(*n* = 57,979)		(*n* = 40,908)		(*n* = 45,505)		*p*-Value ^a^
Age (years)									
20–29	10,168	(14.91)	6833	(11.79)	4399	(10.75)	5425	(11.92)	<0.0001
30–39	14,255	(20.9)	10,030	(17.3)	6860	(16.77)	8456	(18.58)	
40–49	15,007	(22.01)	12,048	(20.78)	8577	(20.97)	10,027	(22.03)	
50–59	11,835	(17.36)	11,373	(19.62)	7787	(19.04)	8393	(18.44)	
≥60	16,927	(24.82)	17,695	(30.52)	13,285	(32.48)	13,204	(29.02)	
Gender									
Male	31,968	(46.88)	27,327	(47.13)	19,281	(47.13)	21,660	(47.6)	<0.0001
Female	36,224	(53.12)	30,652	(52.87)	21,627	(52.87)	23,845	(52.4)	
Marital status									
Married	48,000	(70.39)	41,399	(71.4)	29,916	(73.13)	33,108	(72.76)	<0.0001
Divorced/windowed	7933	(11.63)	8202	(14.15)	5942	(14.53)	6147	(13.51)	
Never married	12,259	(17.98)	8378	(14.45)	5050	(12.34)	6250	(13.73)	
Education									
Elementary or less	13,479	(19.77)	16,302	(28.12)	12,900	(31.53)	12,925	(28.4)	<0.0001
Middle-High school	29,050	(42.6)	25,622	(44.19)	17,501	(42.78)	19,597	(43.07)	
College or higher	25,663	(37.63)	16,055	(27.69)	10,507	(25.68)	12,983	(28.53)	
Monthly income (1000 KRW)									
Q1 (≤1000)	13,612	(19.96)	16,060	(27.7)	11,961	(29.24)	12,690	(27.89)	<0.0001
Q2 (1001–2000)	15,649	(22.95)	15,012	(25.89)	10,124	(24.75)	10,819	(23.78)	
Q3 (2001–3500)	15,397	(22.58)	12,823	(22.12)	8356	(20.43)	9691	(21.3)	
Q4 (≥3501)	23,534	(34.51)	14,084	(24.29)	10,467	(25.59)	12,305	(27.04)	
Job categories									
White-collar	16,048	(23.53)	9863	(17.01)	6548	(16.01)	7926	(17.42)	<0.0001
Pink-collar	9537	(13.99)	7705	(13.29)	5092	(12.45)	5682	(12.49)	
Blue-collar	15,627	(22.92)	18,083	(31.19)	13,918	(34.02)	14,457	(31.77)	
Military	262	(0.38)	255	(0.44)	224	(0.55)	302	(0.66)	
No economic act	26,718	(39.18)	22,073	(38.07)	15,126	(36.98)	17,138	(37.66)	
Cigarette smoking									
Current smoker	16,348	(23.97)	14,161	(24.42)	9871	(24.13)	11,120	(24.44)	<0.0001
Former smoker	9558	(14.02)	7955	(13.72)	5280	(12.91)	6466	(14.21)	
Never smoker	42,286	(62.01)	35,863	(61.86)	25,757	(62.96)	27,919	(61.35)	
Alcohol drinking									
Drinker	36,500	(53.53)	29,198	(50.36)	19,521	(47.72)	23,239	(51.07)	<0.0001
Non-drinker	31,692	(46.47)	28,781	(49.64)	21,387	(52.28)	22,266	(48.93)	
Self-reported stress									
High	19,877	(29.15)	14,966	(25.81)	9939	(24.3)	11,599	(25.49)	<0.0001
Low	48,315	(70.85)	43,013	(74.19)	30,969	(75.7)	33,906	(74.51)	
Moderate activity									
NO	50,069	(73.42)	40,081	(69.13)	28,657	(70.05)	31,416	(69.04)	<0.0001
YES (min)									
Q1 (≤60)	4637	(6.8)	3974	(6.85)	2508	(6.13)	2903	(6.38)	
Q2 (61–89)	5384	(7.9)	4435	(7.65)	2993	(7.32)	3718	(8.17)	
Q3 (90–169)	4389	(6.44)	4010	(6.92)	2853	(6.97)	3433	(7.54)	
Q4 (≥170)	3713	(5.44)	5479	(9.45)	3897	(9.53)	4035	(8.87)	

^a^ *p*-Value was calculated by the chi-square test.

**Table 2:** Odds ratio (95% confidence interval) of hyperlipidemia against the quartiles of parks and green areas.

Parks and Green Area (m^2^ per Capita)	Unadjusted Model		Adjusted Model					
			Model 1		Model 2		Model 3	
Hyperlipidemia, physician diagnose								
Quartile 1 (≤14.90)	1.30	(1.23–1.37)	1.32	(1.25–1.40)	1.32	(1.26–1.40)	1.23	(1.17–1.29)
Quartile 2 (14.90–22.40)	1.11	(1.04–1.17)	1.07	(1.01–1.13)	1.07	(1.01–1.14)	1.03	(0.98–1.09)
Quartile 3 (22.41–33.30)	1.02	(0.96–1.09)	0.99	(0.92–1.05)	0.99	(0.93–1.06)	0.98	(0.92–1.04)
Quartile 4 (≥33.31)	Reference		Reference		Reference		Reference	
*Trend for p*	<0.0001		<0.0001		<0.0001		<0.0001	
Hyperlipidemia, current treatment								
Quartile 1 (≤14.90)	1.46	(1.35–1.58)	1.52	(1.40–1.64)	1.52	(1.41–1.65)	1.45	(1.35–1.56)
Quartile 2 (14.90–22.40)	1.24	(1.15–1.35)	1.19	(1.10–1.29)	1.19	(1.11–1.29)	1.19	(1.11–1.29)
Quartile 3 (22.41–33.30)	1.12	(1.02–1.22)	1.07	(0.98–1.17)	1.07	(0.99–1.18)	1.08	(1.00–1.18)
Quartile 4 (≥33.31)	Reference		Reference		Reference		Reference	
*Trend for p*	<0.0001		<0.0001		<0.0001		<0.0001	

Model 1 was adjusted by age, sex, marriage, education, monthly income, and job categories; Model 2 was further adjusted for smoking and alcohol; Model 3 was further adjusted for a history of diabetes, body mass index, self-reporting stress and moderate physical activity.

**Table 3:** Odds ratio (95% CI) of hyperlipidemia by the quartiles of parks and green areas (Moderate physical activity NO vs. YES).

Park and Green Areas (m^2^ per Capita)	Moderate Physical Activity (NO)		Moderate Physical Activity (YES) ^a^	
	Odds Ratio (95% CI)		Odds Ratio (95% CI)	
Hyperlipidemia, physician diagnose				
Quartile 1 (≤14.90)	1.25	(1.18–1.33)	1.19	(1.08–1.30)
Quartile 2 (14.90–22.40)	1.08	(1.01–1.15)	0.95	(0.86–1.04)
Quartile 3 (22.41–33.30)	1.00	(0.93–1.07)	0.93	(0.84–1.03)
Quartile 4 (≥33.31)	Reference		Reference	
*Trend for p*	<0.0001		<0.0001	
Hyperlipidemia, current treatment				
Quartile 1 (≤14.90)	1.49	(1.37–1.62)	1.35	(1.18–1.55)
Quartile 2 (14.90–22.40)	1.24	(1.14–1.35)	1.09	(0.95–1.25)
Quartile 3 (22.41–33.30)	1.10	(1.00–1.21)	1.05	(0.91–1.23)
Quartile 4 (≥33.31)	Reference		Reference	
*Trend for p*	<0.0001		<0.0001	

^a^ Adjusted by age, sex, marriage, education, monthly income, job categories, smoking, alcohol, self-reporting stress, a history of diabetes, and body mass index. CI, confidence interval.
